# Open and closed loop differential target multiplexed spinal cord stimulation in patients with persistent spinal pain syndrome type II: protocol for a one year European multicenter cohort study (ENDLESS)

**DOI:** 10.3389/fpain.2026.1922595

**Published:** 2026-08-19

**Authors:** Lisa Goudman, Kliment Gatzinsky, Vivek Mehta, Kavita Poply, Ashish Gulve, Iris Smet, Paul Murphy, Dirk Rasche, Georgios Matis, Jan Willem Kallewaard, José Manuel Trinidad, Pasquale De Negri, Philippe Rigoard, ENDLESS consortium, Maarten Moens

**Affiliations:** 1 STIMULUS research group, Vrije Universiteit Brussel, Brussels, Belgium; 2 Department of Neurosurgery, Universitair Ziekenhuis Brussel, Brussels, Belgium; 3 Cluster Neurosciences, Center for Neurosciences (C4N), Vrije Universiteit Brussel, Brussels, Belgium; 4 Department of Physiotherapy, Pain in Motion (PAIN) Research Group, Human Physiology and Anatomy, Faculty of Physical Education and Physiotherapy, Vrije Universiteit Brussel, Brussels, Belgium; 5 Research Foundation—Flanders (FWO), Brussels, Belgium; 6 Department of Neurosurgery, Sahlgrenska University Hospital, Göteborg, Sweden; 7 Barts Neuromodulation Centre, St. Bartholomew's Hospital, London, United Kingdom; 8 Department of Pain Medicine, The James Cook University Hospital, Middlesbrough, United Kingdom; 9 Multidisciplinary Pain Center, Vitaz Ziekenhuis, Sint-Niklaas, Belgium; 10 Pain Medicine, St Vincent's University Hospital, Dublin, Ireland; 11 Department of Neurosurgery, University of Lübeck, Lübeck, Germany; 12 Interventional Pain Management, Spasticity Neuromodulation Unit, Hygeia Hospital, Athens, Greece; 13 Anesthesiology and Pain Medicine, Amsterdam University Medical Centers, Amsterdam, Netherlands; 14 Anesthesiology and Pain Medicine, Rijnstate Ziekenhuis, Velp, Netherlands.; 15 Anesthesiology, Reanimation and Pain Management, Universidad de Cadiz, Cadiz, Spain; 16 AORN S. Anna S. Sebastiano, Caserta, Italy; 17 PRISMATICS Lab (Predictive Research and Innovative Strategies for Medical & Advanced Technology Integration in Care Systems), INSERM 1402, Poitiers University Hospital, Poitiers, France; 18 Department of Spine Surgery & Neuromodulation, Poitiers University Hospital, Poitiers, France; 19 Department of Radiology, Universitair Ziekenhuis Brussel, Brussels, Belgium

**Keywords:** chronic neuropathic pain, closed-loop stimulation, differential target multiplexed spinal cord stimulation, ecological momentary assessment, evoked compound action potentials, neuromodulation, persistent spinal pain syndrome type 2, spinal cord stimulation

## Abstract

Differential target multiplexed spinal cord stimulation (DTM SCS) is a stimulation paradigm for chronic pain management that aims to modulate neuron-glial interactions. In parallel, closed-loop SCS systems using evoked compound action potential (ECAP) sensing have been introduced to adjust stimulation output in response to measured neural activation. Although clinical evidence for DTM SCS has expanded, data remain limited for patients with Persistent Spinal Pain Syndrome Type 2 (PSPS-T2), particularly regarding ECAP-controlled closed-loop DTM SCS and its implementation in routine European practice. ENDLESS aims to investigate the feasibility, safety, and longitudinal clinical outcomes associated with open-loop and closed-loop DTM SCS in patients with PSPS-T2 through a longitudinal European multicentre cohort study. ENDLESS is a prospective multicentre cohort study including at least 200 patients, with follow-up until 12 months after implantable pulse generator activation. Outcomes will be assessed at baseline and at 1, 6 and 12 months after DTM SCS initiation. The primary effectiveness endpoint is pain intensity, measured using the Visual Analogue Scale through ecological momentary assessment. Secondary effectiveness outcomes include pain intensity with and without medication use, negative affect, disability, health-related quality of life, pain medication use, clinical holistic responder status, patient impression of change, work status, pain catastrophising, symptoms of anxiety and depression, patient unwanted stimulation awareness and patient expectations. In a subset of patients, sleep, activity patterns and RR-intervals will be assessed using actigraphy. Device-derived outcomes will include time spent in different body postures, technical implantable pulse generator details, battery consumption, stimulation parameters, programming information and ECAP-related data when closed-loop DTM SCS is used. Technical issues, adverse events, serious adverse events and the proportion of successful DTM SCS trial periods will also be documented. Longitudinal mixed models will be used to evaluate changes in effectiveness outcomes over time.

**Trial registration number:**

ClinicalTrials.gov identifier NCT07211308.

## 1 Introduction

Persistent Spinal Pain Syndrome Type 2 (PSPS-T2) describes persistent or recurrent spinal pain following one or more surgical procedures on the spinal axis and is now preferred to older terminology such as failed back surgery syndrome (1, 2). PSPS-T2 comprises a clinically heterogeneous population in whom axial and radicular pain may coexist and may be influenced by anatomical, neuropathic, psychological, social, and treatment-related factors. This heterogeneity is relevant to spinal cord stimulation (SCS), because clinical outcomes depend not only on the stimulation paradigm but also on patient selection, expectations, pain phenotype, psychosocial characteristics, programming, and the organisation of follow-up care (3, 4). Conventional tonic SCS typically delivers stimulation at 50–60 Hz and may produce paresthesia in the painful area. However, changes in posture, movement, and other physiological factors may alter the relationship between the implanted lead and the spinal cord, resulting in variations in perceived stimulation and neural activation (5–7).

Differential Target Multiplexed SCS (DTM SCS) is a stimulation paradigm that combines multiple charge-balanced electrical signals with different frequencies, pulse widths, amplitudes, and electrode configurations. These signals are delivered through different stimulation fields with the aim of modulating neuronal and glial processes involved in chronic pain (8, 9). Preclinical studies have shown that multiplexed charge-balanced pulsed signals can reduce mechanical hypersensitivity in animal models of neuropathic pain and may modulate cell-specific transcriptomic profiles related to microglia, astrocytes, oligodendrocytes and neurons (9–11). These findings support the hypothesis that DTM SCS may influence neuroglial interactions and rebalance cellular processes involved in chronic pain modulation (12). Randomised clinical studies have subsequently demonstrated favourable clinical outcomes with DTM SCS in selected populations with chronic back and leg pain (13, 14).

Randomised controlled trials remain essential for evaluating treatment efficacy, but they do not fully describe how SCS is implemented across routine European clinical practice. Access criteria, duration and interpretation of trial stimulation, reimbursement rules, available programming resources and follow-up pathways may differ between countries and centers (15). These factors may influence trial success, time to optimization, programming decisions, patient satisfaction, battery consumption, and long-term continuation of therapy. Prospective observational research can therefore complement controlled trials by evaluating treatment delivery and longitudinal clinical outcomes in broader patient populations under routine-care conditions. The previous DETECT study established a framework for evaluating DTM SCS in a multicentre cohort, while ENDLESS extends this approach to a larger European population and incorporates both open-loop and closed-loop stimulation (16).

A related technological development is the integration of evoked compound action potential (ECAP) sensing into SCS systems. Conventional open-loop SCS delivers stimulation at the programmed output without continuously measuring the resulting neural response. ECAPs are electrophysiological signals generated by the synchronous activation of spinal cord axons in response to stimulation and can be recorded from the implanted epidural leads (17, 18). Their amplitude varies with the degree of neural activation and may change when posture or movement alters the distance between the lead and the spinal cord (18, 19). ECAP-informed closed-loop systems use the measured neural response as feedback to adjust stimulation amplitude automatically within clinically programmed limits. This approach is intended to reduce variations in neural activation associated with posture and movement while allowing contemporary stimulation paradigms, including multiplexed stimulation, to be delivered using feedback-informed control (20).

Evaluating DTM SCS in routine practice also requires outcome measures capable of capturing the fluctuating and multidimensional nature of chronic pain. Pain intensity in chronic pain fluctuates within and between days and may be influenced by activity, affect, medication use and context (21). Ecological momentary assessment (EMA) can reduce recall bias by repeatedly assessing symptoms in the patient's natural environment rather than relying only on retrospective ratings at clinic visits (22). By combining EMA-based pain and medication data, validated questionnaires, work participation, patient-reported unwanted stimulation awareness, and implantable pulse generator (IPG)-derived technical data, ENDLESS aims to evaluate not only whether DTM SCS is associated with clinical improvement, but also how therapy is used, optimized and experienced during the first year after implantation.

ENDLESS is therefore a prospective European multicentre cohort study designed to investigate the feasibility, effectiveness and safety of open-loop and closed-loop DTM SCS in patients with PSPS-T2 over 12 months. ENDLESS is designed to evaluate longitudinal clinical outcomes, feasibility, safety and implementation of DTM SCS under routine-care conditions. Therefore, it will complement existing randomized and national cohort evidence by focusing on generalisability, programming feasibility, ECAP-informed delivery, patient-centred outcomes and real-world implementation across multiple European centres. It is not designed to establish stimulation-specific efficacy or to distinguish the physiological effects of stimulation from placebo effects, patient expectations, clinical attention, natural symptom fluctuations or other contextual components of the treatment pathway.

## 2 Materials and equipment

The ENDLESS protocol uses a combination of clinical, patient-reported and device-derived data sources. DTM SCS will be delivered using IPGs capable of open-loop and/or closed-loop DTM SCS delivery. ECAP-related information will be collected when available. Patient-reported outcomes will be collected electronically using Qualtrics, and clinical, safety, feasibility and device-related data will be entered in REDCap. Ecological momentary assessment of pain, medication use and negative affect will be performed using m-Path. In a subset of patients, actigraphy data will be collected using Garmin Venu series devices. Device-derived information will include IPG output, stimulation parameters, programming data, battery performance, recharge behaviour, JavaScript Object Notation (JSON) files and session reports. Statistical analyses will be performed using RStudio and SAS.

## 3 Methods

### 3.1 Study design

ENDLESS is a prospective, observational, European multicentre cohort study designed to evaluate DTM SCS delivered in open-loop or closed-loop mode in patients with PSPS-T2. The study will be conducted in academic and regional hospitals across Europe and the United Kingdom. The choice between open-loop and closed-loop DTM SCS will not be assigned by the study protocol and will be determined according to routine clinical practice, and clinician judgement. The protocol does not require a one-centre–one-stimulation-mode approach. Participating centres are experienced in SCS implantation and follow-up, and were selected based on clinical expertise, availability of DTM SCS, and the expected ability to recruit and monitor patients with PSPS-T2 in routine care. An overview of participating study sites is available on ClinicalTrials.gov under identifier NCT07211308.

Recruitment started on 9 December 2025 and will continue until at least 200 patients have been included. As ENDLESS is designed to reflect routine clinical practice, no randomisation or blinding will be applied. All enrolled patients will receive DTM SCS as part of standard clinical care according to local practice, device availability and country-specific reimbursement regulations.

### 3.2 Eligibility criteria

Patients will be eligible for inclusion if they are adults scheduled for SCS implantation with DTM SCS as the intended primary stimulation paradigm. The study population will consist of patients with PSPS-T2 and chronic back and leg pain, and may also include patients with chronic neck and arm pain after previous spinal surgery.

Patients must meet all of the following inclusion criteria:

- diagnosis of PSPS-T2, defined as persistent or recurrent spinal pain after one or more surgical procedures on the spinal axis, performed to relieve axial spinal pain, radicular pain or a combination of both, with insufficient treatment effect (23);
- chronic back/neck pain with leg/arm pain attributed to PSPS-T2 and refractory to best medical treatment;
- pain duration of at least 6 months;
- at least moderate pain intensity at the dominant pain location, defined as a score of 4 or higher on a Numeric Rating Scale or 31 mm or higher on a Visual Analogue Scale;
- planned treatment with DTM SCS as the primary SCS paradigm;
- age of 18 years or older.

Patients will be excluded in case of any of the following:

- expected inability to operate the SCS system adequately;
- History of coagulation disorders, lupus erythematosus, diabetic neuropathy, rheumatoid arthritis or Morbus Bechterew;
- active malignancy;
- addiction to drugs or alcohol, defined as more than 5 units of alcohol per day;
- active disruptive psychiatric disorder or another condition judged by the investigator to interfere with pain perception, adherence to the intervention or ability to complete study assessments;
- immune deficiency, including human immunodeficiency virus infection or use of immunosuppressive therapy;
- life expectancy shorter than 1 year;
- local infection or other skin disorder at the planned incision site;
- pregnancy;
- presence of another implanted active medical device.

No formal hypothesis-based sample-size calculation was performed because ENDLESS is a pragmatic observational cohort study and is not designed to test a prespecified superiority hypothesis between open-loop and closed-loop DTM SCS. A target sample of at least 200 participants was selected *a priori* based on the recruitment experience and feasibility established in the previous DETECT multicentre cohort study, which used the same target sample size, and on the expected recruitment capacity of the participating centres (16). This sample is intended to provide a sufficiently large real-world cohort for the description of longitudinal clinical, feasibility, safety, programming, and device-derived outcomes over 12 months. It should not be interpreted as providing formal statistical power for comparative subgroup analyses or for detecting uncommon adverse events. Comparisons between stimulation modes and other subgroup analyses will therefore be exploratory and interpreted in relation to the achieved subgroup sizes, confidence intervals, and extent of missing data.

### 3.3 Interventions

Potentially eligible patients will be informed about the study by the treating neurosurgeon, anaesthesiologist, pain physician or another delegated member of the local study team. Patients who are interested in participation will be screened against the eligibility criteria. Eligible patients will receive oral and written study information and will have the opportunity to ask questions before signing informed consent. Baseline assessments will be completed before the SCS trial.

After baseline evaluation, patients will undergo an SCS trial according to local clinical practice and the reimbursement rules of the participating country. Lead placement will be performed according to routine clinical practice. The ENDLESS protocol does not mandate a specific lead type, number of leads, or fixed vertebral implantation level. These procedural characteristics will be determined by the treating physician according to the anatomical distribution of pain, previous spinal surgery, patient anatomy, intraoperative findings, and local practice. The lead type, including whether a percutaneous or surgical lead is used where applicable, the number of leads, their final anatomical position, and other relevant implantation details will be recorded for each participant.

IPG implantation will be performed after a successful trial, defined as at least 50% pain reduction or according to country-specific reimbursement criteria when these differ. Patients will receive an Inceptiv™ IPG capable of delivering DTM SCS in open-loop and/or closed-loop mode.

DTM SCS uses separate charge-balanced stimulation programmes that differ in frequency, pulse width, amplitude, and potentially electrode configuration. These programmes are referred to as the “base” and “prime” components and should not be interpreted as frequency-modulated currents. The base component generally uses a frequency between 20 and 200 Hz, whereas the prime component uses a frequency between 200 and 1,200 Hz. The programmes are combined within a multiplexed stimulation pattern, with or without cycling (13, 24).

The base and prime components may use different cathode–anode configurations to generate different stimulation fields along the implanted leads. Electrode configurations are individualised according to lead position, pain distribution, patient comfort, clinical response, and physician judgement. For open-loop DTM SCS, the initial settings used in ENDLESS will generally consist of a base programme at 50 Hz and 200 µs and a prime programme starting at 300 Hz and 170 µs (13). Amplitude and subsequent programming adjustments will be individualised within predefined clinical safety limits.

For closed-loop DTM SCS, the same therapeutic DTM framework is used, with the addition of ECAP sensing to permit automatic adjustment of stimulation amplitude according to the measured neural response. In the closed-loop DTM configuration, the 50 Hz component functions both as a therapeutic component and as the stimulation signal used to elicit ECAPs. The ECAP generated by this component provides the feedback signal for the closed-loop algorithm (24).

Patient-specific ECAP reaction and recovery thresholds are established during clinical programming. When the measured ECAP amplitude exceeds the reaction threshold, indicating increased neural activation, the system automatically reduces stimulation amplitude. When ECAP amplitude subsequently falls below the recovery threshold, stimulation amplitude is increased toward the programmed setting. When ECAPs are consistently measurable at rest, the system adjusts stimulation amplitude to keep the measured neural response within the patient-specific threshold range. These adjustments are performed repeatedly during stimulation and are intended to reduce variations in neural activation caused by posture, movement, and changes in the distance between the lead and spinal cord (24).

In multiplexed DTM SCS, the ECAP elicited by the 50-Hz component is used to regulate the stimulation amplitudes of the associated programmes. Changes in the amplitude of the additional programme or programmes are made proportionally to changes in the ECAP-generating component. Frequency, pulse width, electrode configuration, and programme sequencing are not automatically modified by the ECAP feedback algorithm.

Patients may adjust stimulation within limits predefined by the treating clinician. In fixed-output open-loop mode, stimulation amplitude remains at the programmed level until adjusted by the patient or clinician. If posture-responsive AdaptiveStim is enabled, amplitude may instead change automatically between clinician-programmed posture-specific settings; these adjustments are accelerometer-based rather than ECAP-based. When the closed-loop feature is active and a usable ECAP signal is available, the system may automatically increase, decrease, or hold stimulation amplitude in response to the measured ECAP signal. Patient-controlled adjustment and automatic ECAP-based regulation therefore represent distinct components of therapy management.

The intended daily stimulation schedule will be determined according to routine clinical practice and individual patient needs. Programme use, stimulation duration where available, interruptions in therapy, stimulation parameters, programming changes, battery consumption, and recharge behaviour will be documented throughout follow-up. For patients receiving closed-loop DTM SCS, ECAP-related information and available device-derived technical data will also be collected to evaluate the feasibility of ECAP-informed stimulation delivery in routine clinical practice.

Patients who do not wish to continue DTM SCS after an adequate trial or during follow-up may be switched to other SCS settings according to clinical judgement and local practice. The timing and reason for any change in stimulation strategy will be recorded. Follow-up will continue according to the ENDLESS protocol, regardless of whether DTM SCS is continued or another stimulation setting is used.

### 3.4 Outcomes

#### 3.4.1 Effectiveness outcomes

The outcome measures are described below, while their timing and collection frequency are summarised in Table 1.

**Table 1:** Overview of outcome measurements at each ENDLESS study visit.

Outcome domain	Outcome measurement	Measurement tool/source	Collection frequency/window	Baseline screening V0	1 month V1	6 months V2	12 months V3
Effectiveness	Current pain intensity	VAS through EMA	Three prompts daily for seven consecutive days at each assessment period	✓	✓	✓	✓
	Average pain intensity	NRS	Once per applicable assessment period	✓	✓	✓	✓
	Medication use	EMA	Three prompts daily for seven consecutive days at each assessment period	✓	✓	✓	✓
	Negative affect	EMA	Three prompts daily for seven consecutive days at each assessment period	✓	✓	✓	✓
	Disability	ODI/NDI	Once per applicable assessment period	✓	✓	✓	✓
	Health-related quality of life	EQ-5D-5L	Once per applicable assessment period	✓	✓	✓	✓
	Impression of change	PGIC	Once per applicable assessment period	—	✓	✓	✓
	Sleep, activity patterns and RR-intervals	Actigraphy subset	Continuous recording for seven consecutive days in the subset	✓	✓	✓	✓
	Holistic responder status	Composite measure	Derived after completion of data collection using outcomes obtained at each follow-up assessment	—	✓	✓	✓
	Pain catastrophizing	PCS	Once per applicable assessment period	✓	✓	✓	✓
	Anxiety and depression	HADS	Once per applicable assessment period	✓	✓	✓	✓
	Work status	Self-developed questionnaire	Once per applicable assessment period	✓	—	✓	✓
	Patient expectations and fulfilment	Open question	After last assessment period	✓	—	—	✓
	Patient unwanted stimulation awareness	Self-developed questionnaire	Once per applicable assessment period	—	✓	✓	✓
	Time spent in body postures	IPG output	Extracted at visits for the preceding follow-up interval	—	✓	✓	✓
Safety	Adverse events and serious adverse events	Physician reporting	Recorded continuously and reviewed at each follow-up	—	✓	✓	✓
Feasibility	Proportion of successful DTM SCS trials	Investigator/device reporting		—	✓	✓	✓
	Technical IPG details, including ECAP-related data	IPG output	Extracted at visits for the preceding follow-up interval	—	✓	✓	✓
	DTM SCS stimulation parameters	IPG output	Extracted at visits for the preceding follow-up interval	—	✓	✓	✓
	Battery consumption/recharge frequency	IPG output	Extracted at visits for the preceding follow-up interval	—	✓	✓	✓
	Technical issues with DTM SCS programming	IPG output/physician reporting	Extracted at visits for the preceding follow-up interval	—	✓	✓	✓

**Abbreviations:** DTM, differential target multiplexed; ECAP, evoked compound action potential; EMA, ecological momentary assessment; EQ-5D-5L, EuroQol five-dimension, five-level questionnaire; HADS, hospital anxiety and depression scale; IPG, implantable pulse generator; NDI, neck disability index; NRS, numeric rating scale; ODI, Oswestry disability index; PCS, pain catastrophizing scale; PGIC, patient global impression of change scale; SCS, spinal cord stimulation; V, visit; VAS, visual analogue scale.

The primary effectiveness outcome measure is current pain intensity, measured electronically with the Visual Analogue Scale (VAS), ranging from 0 (no pain) to 100 (maximal pain). Separate ratings will be obtained for low back or neck pain and for leg or arm pain. Pain intensity will be assessed using EMA during seven-day assessment periods. Participants will receive three prompts per day at randomly selected times within the following intervals: 07:30–10:30, 12:00–15:00, and 18:00–21:00. EMA will be administered using the m-Path platform. The validity and reliability of EMA-based pain assessment have been supported in patients with chronic pain (25).

Secondary effectiveness outcomes will include the following

1. Average pain intensity: Average pain intensity in the lower back or neck and leg or arm during the preceding seven days will be assessed using an 11-point Numeric Rating Scale. Participants will provide ratings of pain intensity both with and without concurrent analgesic use (26).
2. Medication use and momentary pain-related experiences**:** During each EMA period, participants will report whether they have taken pain medication during the preceding hours and, where applicable, the medication type and dose. Medication use will be summarised using the Medication Quantification Scale, version III or a later version. EMA will also assess pain-related interference, momentary pain-related cognitions, and negative affect (27). Pain-related interference will be assessed using the question, “To what degree did your pain interfere with your being physically active?” Momentary pain-related cognitions will be evaluated by asking participants to what degree they feared that their pain might worsen, continued thinking about how much it hurt, or experienced the pain as awful and overwhelming. Negative affect will be assessed by asking participants to rate the extent to which they felt anxious, on edge, uneasy, sad, helpless, discouraged, irritated, annoyed, or angry. These items will be rated on a nine-point scale.
3. Disability: Disability will be assessed using the Oswestry Disability Index for participants with predominantly low back pain or the Neck Disability Index for participants with predominantly neck pain (28, 29).
4. Health-related quality of life: Health-related quality of life will be evaluated using the EuroQol five-dimension, five-level questionnaire (30).
5. Patient-perceived change: Overall change since initiation of DTM SCS will be assessed using the seven-point Patient Global Impression of Change scale (31).
6. Clinical holistic responder status: Clinical holistic responder status will be derived after completion of data collection using a predefined multicomponent approach combining pain intensity, medication use, disability, health-related quality of life, and patient-perceived change (32).
7. Work status: Work participation will be evaluated using a study-specific questionnaire (33, 34). follow-up, they will report whether and when they resumed professional activities, whether they returned full-time or part-time, and whether changes in occupation or job content were required.
8. Patient expectations and fulfilment: Before treatment, participants will describe their expectations of DTM SCS using an open-ended question. At the final assessment, they will be asked to reflect on the extent to which these expectations were fulfilled.
9. Pain catastrophising: Pain catastrophising will be assessed using the Pain Catastrophizing Scale (35, 36).
10. Symptoms of anxiety and depression: Symptoms of anxiety and depression will be assessed using the 14-item Hospital Anxiety and Depression Scale (37).
11. Unwanted stimulation awareness: Awareness of unwanted stimulation will be assessed using a study-specific questionnaire
12. Body posture: When the relevant device feature is activated, time spent in different body postures will be obtained from the IPG. Posture data will be extracted for the last 30 days for the periods from IPG activation to 1 month, from 1 to 6 months, and from 6 to 12 months after IPG activation.
13. Activity, sleep, and heart rate variability: In a subset of participants recruited in Belgium, activity and sleep patterns will be assessed using a Garmin Venu series device. Beat-to-beat R–R intervals will also be collected, from which time-domain, frequency-domain, and non-linear heart rate variability parameters will be derived (38). Participants will wear the device for seven consecutive days, aligned with the corresponding EMA period where feasible. Wearable-device data will be collected through the Labfront platform.

#### 3.4.2 Safety related outcome

The primary safety outcome will be the occurrence of adverse events and serious adverse events during the study. Any unfavourable change in health, or the onset or worsening of an undesirable sign, symptom or medical condition after enrolment, will be recorded as an adverse event (AE), irrespective of whether it is considered related to the intervention. All AEs will subsequently be classified according to severity and their relationship to the device and/or procedure.

A serious adverse event (SAE) will be defined as any untoward medical occurrence, whether or not considered related to the investigational intervention, that results in death, is life-threatening, requires hospitalisation or prolongation of existing hospitalisation, results in persistent or significant disability or incapacity, constitutes a congenital anomaly or birth defect, requires intervention to prevent permanent impairment or damage, or is considered an important medical event. Hospitalisations related to elective or previously planned procedures for pre-existing conditions that have not worsened after study enrolment will not be considered SAEs.

SAEs will be reported to the sponsor and handled in accordance with applicable national legislation and local ethics committee requirements. The investigator will be responsible for assessing and reporting safety events throughout the study. Both AEs and SAEs must be reported to the sponsor and principal investigator in a timely manner according to local requirements.

#### 3.4.3 Feasibility related outcome

Feasibility will be assessed by determining the proportion of patients with a successful DTM SCS trial. Technical feasibility will be evaluated by documenting technical issues related to DTM SCS, battery consumption and recharge frequency. Programming information will be collected for the different stimulation groups, including the percentage use of each group. Clinically effective stimulation parameters, including frequency, pulse width and amplitude, will be identified at each post-implantation visit.

Additional technical information will be obtained from the IPG. For patients treated with closed-loop DTM SCS, in-clinic ECAP streams will be collected to evaluate ECAP signal stability through JSON files. At-home SCS device use, programming and IPG battery performance will be obtained from Medtronic data reports and analysed as part of the feasibility evaluation.

**Figure 1:**
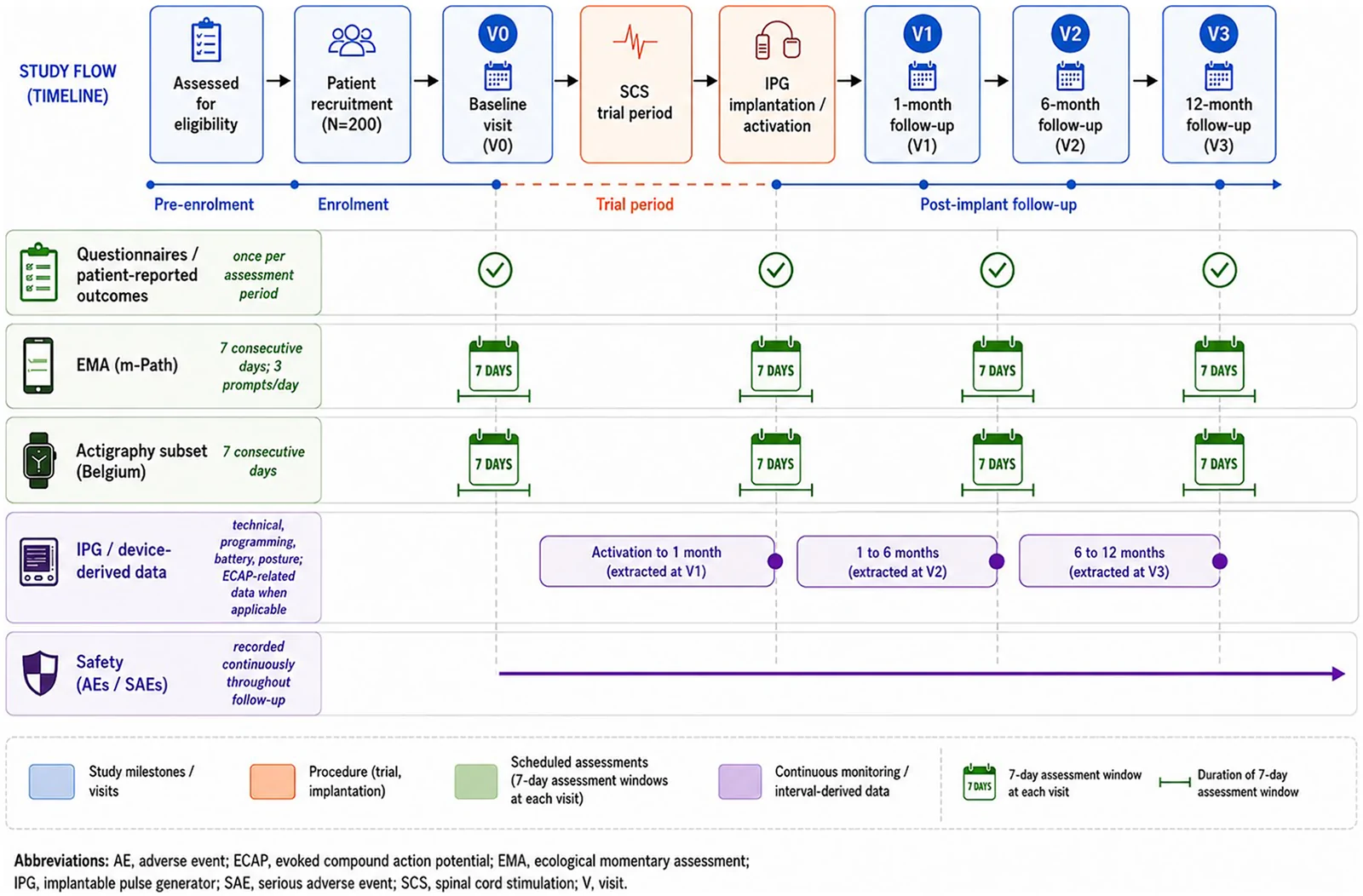
Patient flow chart. Overview of screening, recruitment, baseline assessment, SCS trial, IPG implantation and follow-up visits at 1, 6 and 12 months after IPG activation with the set of outcome measures.

The overall study flow is shown in Figure 1. If a patient does not attend a scheduled outpatient follow-up visit, the study team will attempt to contact the patient by telephone. When the patient agrees, any assessments that can be completed remotely will be collected by phone.

### 3.5 Patient and public involvement

The selection of questionnaires was informed by previous work identifying treatment goals in patients before SCS implantation through qualitative interviews with independent researchers (39), as well as by studies exploring outcomes that patients with chronic pain aim to achieve (40). In addition, experience gained during the previous DETECT study was used to refine the assessment battery for ENDLESS (16). Questionnaires that were perceived as difficult or burdensome by patients were reconsidered, and the outcome set was adapted where possible to improve feasibility and acceptability while maintaining clinically relevant assessment domains. Patient support groups will be offered the opportunity to discuss the study findings after completion of the study. The results of ENDLESS may further support patient and public involvement by identifying outcomes and assessment methods that are meaningful and feasible for patients undergoing DTM SCS.

### 3.6 Data management

Study data will be collected and managed using secure web-based data collection systems. Qualtrics will be used for patient-reported outcome measures. Patients will complete questionnaires by accessing a study-specific web link or by scanning a quick-response code that directs them to the relevant assessment. REDCap will be used as the electronic case report form for clinical, safety, feasibility and device-related data entered by the investigator or delegated study personnel.

Each participant will be identified by a unique study code consisting of the centre identification number and an individual patient number assigned by the investigator. Directly identifiable personal information will be stored separately from study data where possible, and analyses will be performed on pseudonymised datasets.

The following source data will be generated during the study: medical history obtained from the patient's medical file to assess eligibility; signed informed consent forms; questionnaire results; personal health-related study data; safety reports; SCS programming parameters and device-derived data, including comma-separated value files, JSON files and IPG session reports; and statistical analysis scripts in R and SAS-readable formats.

For EMA and Garmin data collection, the Labfront and m-Path platforms will be used. Data will be encrypted during transmission, and Labfront data will be archived using Amazon Web Services S3 Glacier. The m-Path system was developed according to privacy-by-design principles. Both platforms will be used in accordance with the European Union General Data Protection Regulation and applicable institutional data-protection requirements (41).

Source documents will be completed at the time of the participant's visit or as soon as possible thereafter. Participating sites will retain relevant source documents and centre-specific essential documents in the Investigator Site File. Study-related documents and trial data will be archived securely in accordance with International Council for Harmonisation Good Clinical Practice guidelines and applicable national regulations. Measures will be taken to prevent accidental loss, premature destruction or unauthorised access. Trial data will not be destroyed without the sponsor's agreement. Patient identification codes will be retained for at least 25 years after completion or discontinuation of the study. The investigator will allow study-related monitoring, audits, Ethics Committee review and regulatory inspections by providing direct access to relevant source data and documents.

### 3.7 Data analysis

Data processing and statistical analyses will be performed on pseudonymised datasets after completion of follow-up, data monitoring and data cleaning. Analyses will be conducted by the project biostatistician using RStudio and SAS. Patient flow throughout the study will be presented in a flow diagram. Baseline demographic and clinical characteristics will be summarised descriptively for the total study population and, where relevant, by stimulation mode or other clinically meaningful subgroups.

Given the longitudinal design of ENDLESS, repeated measurements within patients will be accounted for in the statistical analyses. The primary outcome, pain intensity up to 12 months after IPG activation, will be analysed using longitudinal mixed models for the overall cohort. Repeated observations will be modelled within patients, and clustering of patients within recruiting centres will be accounted for, where supported by the data. Comparisons between open-loop and closed-loop DTM SCS will be considered exploratory and will account for relevant baseline characteristics and centre-level clustering. Where feasible, sensitivity analyses will be performed in centres that enrolled patients treated with both stimulation modes. If stimulation mode is completely or nearly completely confounded with recruiting centre, comparative results will be reported descriptively and will not be interpreted as evidence of causal comparative effectiveness.

Secondary outcomes will be analysed according to the type and distribution of the data. Longitudinal mixed models will be used for continuous repeated outcomes, generalized mixed models for categorical or non-normally distributed outcomes, and Tweedie generalized linear models when outcomes show a discrete mass at zero (42). Claims of statistical significance for secondary outcomes will only be made if a statistically significant effect is observed for the primary outcome. If the primary outcome is not statistically significant, analyses of secondary outcomes will be interpreted descriptively and exploratively. Main analyses will be performed using the data as observed. Sensitivity analyses will be conducted to assess the robustness of the findings in relation to the handling of missing data.

In addition to mixed-effect modelling, latent class trajectory modelling (LCTM) will be applied to explore patterns of response over time (43, 44). LCTM allows heterogeneous patient populations to be grouped into more homogeneous clusters based on an unobserved latent variable (45). Random effects can be incorporated to account for individual variation within these clusters (46). This data-driven approach will be used to identify trajectories of clinical response within the ENDLESS cohort, rather than assigning patients to predefined categories based only on baseline patient or clinical characteristics.

Patient-level quality-adjusted life-years (QALYs) will be calculated using EQ-5D-5L index scores. QALYs will be estimated using the area under the curve method, in which utility values are linearly interpolated over the study period and the resulting areas are summed (47). A second QALY estimate will be calculated by extrapolating the baseline EQ-5D-5L index value over 12 months, representing the expected QALY without additional treatment (34). Incremental QALYs will be calculated by subtracting the baseline-extrapolated QALY from the QALY observed during DTM SCS over the 12-month follow-up period.

### 3.8 Data monitoring and oversight

The trial management group, consisting of M.M. and L.G., will act as the main decision-making and steering body of the project. A kick-off meeting was organised at the start of the study to establish common working procedures across participating centres. The responsibilities of the trial management group include agreeing on study procedures and management policies, monitoring overall study progress and deliverables, and deciding on major changes to the work programme.

Principal investigators at each recruiting centre will be responsible for patient recruitment and data collection at their own site. The study coordinator at Vrije Universiteit Brussel will regularly review entered data for completeness and consistency and will coordinate data queries when needed. Statistical analyses will be conducted by the project biostatistician using pseudonymised datasets and according to the prespecified statistical analysis strategy. The trial management group will additionally oversee scientific communication, dissemination of study results and exploitation of project outputs. The funder will not have authority to modify the analysis, interpretation or publication of the study findings.

## 4 Anticipated results

ENDLESS is expected to provide pragmatic longitudinal evidence on the feasibility, effectiveness and safety of open-loop and closed-loop DTM SCS in patients with PSPS-T2 across European clinical practice. The study will generate data on pain trajectories, patient-reported outcomes, medication use, work participation, unwanted stimulation awareness, technical programming parameters, ECAP-related information, battery performance and safety events over 12 months. By combining repeated patient-reported assessments with device-derived data, ENDLESS will provide insight into how DTM SCS is delivered, optimized and experienced in routine care. The planned trajectory analyses may also identify clinically relevant response patterns within this heterogeneous patient population.

## 5 Discussion

This protocol describes a pragmatic European cohort study designed to evaluate open-loop and closed-loop DTM SCS in patients with PSPS-T2. The main strength of ENDLESS is its combination of standardized follow-up, ecological momentary assessment, validated patient-reported outcomes and device-derived technical data across multiple countries and clinical settings. This design will allow evaluation of both clinical outcomes and real-world feasibility, including programming characteristics, ECAP-informed delivery, battery performance and patient unwanted stimulation awareness.

The observational design also introduces limitations. As patients will not be randomized and treatment will be delivered according to local clinical practice, causal conclusions regarding comparative effectiveness between open-loop and closed-loop DTM SCS will be limited. The absence of a control group or sham-stimulation condition is an additional limitation. Pain and other patient-reported outcomes may be influenced by expectations, clinical attention, regression to the mean, natural symptom fluctuations and other contextual components of care. Consequently, observed changes cannot be attributed exclusively to the physiological effects of DTM SCS. The study should therefore be interpreted as an evaluation of real-world clinical outcomes and implementation rather than as a controlled assessment of stimulation-specific efficacy. Randomised and, where methodologically and ethically feasible, sham-controlled studies remain necessary to establish causal treatment effects and comparative efficacy. In addition, country-specific reimbursement rules, trial procedures and follow-up pathways may influence treatment implementation and outcomes. Nevertheless, these differences are also clinically relevant, as they reflect the context in which SCS is delivered in routine European practice. ENDLESS is therefore expected to complement existing randomized and national cohort evidence by documenting the implementation, feasibility and patient-centred outcomes of DTM SCS in a real-world PSPS-T2 population.

### 5.1 Ethics considerations

The study protocol was approved by the central Ethics Committee of the Universitair Ziekenhuis Brussel/Vrije Universiteit Brussel (B.U.N.1432024000335), as well as by the Ethics Committees of the participating centres where required. The study will be conducted in accordance with the principles of the Declaration of Helsinki, applicable European and national regulatory requirements, and the current Good Clinical Practice guidelines.

All study data will be handled in accordance with the European Union General Data Protection Regulation (GDPR) and applicable national data protection requirements. Participants will be identified in the study database by a unique participant identification code. Documents submitted to the coordinating centre, sponsor or principal investigator will only contain this study code and will not include directly identifying information.

The key linking participant codes to personal identifying data will be stored locally at each participating site and will only be accessible to authorised site personnel. Names and other directly identifying details will not be entered into the study database. Pseudonymised data will be used for analysis.

## 6 Dissemination plan

Study progress will be communicated regularly to participating centres and relevant stakeholders to support engagement and consistent study conduct. Findings will be disseminated through peer-reviewed scientific publications, presentations at national and international conferences and, where appropriate, professional and social media channels. Study results will also be reported to the relevant regulatory authorities and Ethics Committees, as required.

Publications arising from ENDLESS will follow accepted scientific and academic standards. Authorship will be determined according to the International Committee of Medical Journal Editors (ICMJE) criteria. Publications may be submitted on behalf of the ENDLESS consortium, with investigators from participating sites acknowledged or listed as collaborators according to journal requirements.

After completion of the project, access to pseudonymised study data may be considered under controlled conditions. Any reuse of data will require appropriate approval, including ethical review where applicable, and will be governed by a data use agreement specifying confidentiality obligations, non-disclosure requirements and safeguards for secure data storage.

## ENDLESS consortium

Collaborators in the ENDLESS consortium include: Jean-Pierre Van Buyten, Ali Jerjir, Nina D'hondt, Wout Van Oosterwyck, Mark R van Ooijen, Inge JJ Arnts, N. Koning, Esmee van Leeuwen, Bart Billet, Karel Hanssens, and Ruben De Vos.
